# Evaluating a Web-Based Self-Management Intervention in Heart Failure Patients: A Pilot Study

**DOI:** 10.2196/resprot.5093

**Published:** 2016-06-20

**Authors:** Nazli Bashi, Carol Windsor, Clint Douglas

**Affiliations:** ^1^ School of Nursing Faculty of Health Queensland University of Technology Brisbane Australia

**Keywords:** heart failure, knowledge, patient education, self-care, self-efficacy, self-management, web-based intervention

## Abstract

**Background:**

Web-based interventions may have the potential to support self-care in patients with chronic disease, yet little is known about the feasibility of Web-based interventions in patients with heart failure (HF).

**Objective:**

The objective of our study was to develop and pilot a Web-based self-care intervention for patients with HF.

**Methods:**

Following development and pretesting, we pilot tested a Web-based self-care intervention using a randomized controlled design. A total of 28 participants completed validated measures of HF knowledge, self-care, and self-efficacy at baseline and 1-month follow-up.

**Results:**

Change scores and effect size estimates showed that the mean differences in HF knowledge (*d*=0.06), self-care (*d*=0.32), and self-efficacy (*d*=0.37) were small. Despite email reminders, 7 of 14 participants (50%) of the sample accessed the site daily and 4 of 14 (28%) had no record of access.

**Conclusions:**

Larger randomized controlled trials are needed that attend to all sources of self-efficacy and include more comprehensive educational tools to improve patient outcomes.

## Introduction

Despite improvements in treatment and prevention, chronic heart failure (HF) remains a serious health burden and carries a poor prognosis [1]. Given the complex and progressive nature of HF, interventions are needed that slow disease progression and prevent hospital admissions [2]. One strategy to improve health outcomes in patients with HF is to enhance self-care behavior related to physical activity, nutrition, fluid management, and treatment adherence [3]. Self-care is defined as “a process of maintaining physiological stability by monitoring symptoms, adhering to the treatment regimen (self-care maintenance), and promptly identifying and responding to symptoms (self-care management)” [4]. Self-care education is a critical strategy in empowering HF patients to be informed and actively engaged in monitoring their condition and adjusting treatment accordingly [5].

Although findings are mixed, previous research suggests that Web-based interventions may have the potential to improve clinical outcomes and reduce hospitalizations in patients with chronic diseases including HF [6,7]. Using a Web-based app as a cost-effective intervention might facilitate access to education and improve self-care skills [8]. Yet there are very few clearly conceptualized studies that have evaluated Web-based interventions for HF patients. Two descriptive studies reported high levels of patient satisfaction with educational websites providing information [9] and a daily communication method between HF patients and health care providers [10]. Findings indicated that older HF patients with limited computer skills were willing to engage with Web-based education if provided adequate resources and instruction [9].

Only two controlled studies could be identified that examined the effectiveness of a Web-based HF self-care program. Westlake et al [11] tested a Web-based HF and symptom management education progam specifically designed for older HF patients (≥60 years). In addition to educational modules, patients were encouraged to use the website at home to email a clinical nurse specialist for support, link to video-based Web content, and monitor personal clinical data. A total of 40 patients attending an outpatient HF clinic were recruited to the 12-week Web-based intervention and were compared with 40 age- and sex-matched historical controls who received HF care as usual. Groups were similar at baseline, and results showed a modest benefit for perceived control over health status and mental health quality of life, but not for physical health quality of life.

Another study [12] also tested a Web-based HF self-care program. In addition to usual care, 16 HF patients randomly assigned to the treatment group received a computer with Internet access, as well as basic computer training. The website provided self-care information and videos, access to record and monitor vital signs, health behavior, and HF symptoms daily, and emails from health professionals for feedback on progress and emotional support as needed. It was found that at both 6- and 12-month follow-ups, only the treatment group showed significantly improved HF knowledge, amount of exercise, and quality of life, as well as reduced HF symptoms, blood pressure, and health care utilization [12].

The preliminary findings of the HF studies above, combined with systematic reviews [13,14], suggest that Web-based educational interventions may have a role in supporting HF patients in the community. In this study, we pilot tested a Web-based self-care intervention that provided education and self-monitoring tools for ambulatory HF patients. Our primary aim was to evaluate the feasibility of a Web-based intervention in improving HF patients’ knowledge, self-care, and self-efficacy.

## Methods

### Design

The website development phase involved seeking expert feedback and pretesting the user friendliness and appropriateness of the intervention and outcome measures with HF patients. We recruited an expert panel comprising two cardiologists, four cardiac nurse researchers, and one HF nurse practitioner to advise on the development and content of the website. The software was developed by an information technology team that also provided feedback on the design. We recruited a group of 10 patients with chronic HF visiting a community outpatient HF service to pretest the intervention. During face-to-face meetings, patients provided their feedback and recommendations on the Web-based app content, ease of navigation, and user friendliness. To examine the feasibility of the Web-based HF self-care intervention, we then piloted the intervention using a randomized controlled design over a 1-month period.

### Participants

The pilot sample comprised 29 patients consecutively recruited from a tertiary hospital HF service and a university health clinic located in Brisbane, Australia, between December 2013 and May 2014. Patients were eligible if they were English-speaking adults, had cardiologist- diagnosed class I–III HF according to the New York Heart Association (NYHA) classification and had a left ventricle ejection fraction <40%. Participants were required to have home or mobile Internet access. Exclusion criteria included nursing home residents, severe cognitive impairment, and critical illness.

### Procedure

After providing informed consent at an introductory face-to-face session, participants completed a baseline questionnaire and were randomly assigned to the intervention or control group. Participants in the intervention group were assigned a username and password and were provided basic training on how to use each component of the website. They could then access the website educational modules each day, and record and monitor personal data such as HF-specific symptoms. The intervention group also received a weekly personalized email to improve engagement with the intervention. Both intervention and control groups received usual care from the HF service or clinic, which included comprehensive educational information consisting of topics such as medication, nutrition, exercise, and psychosocial issues. Individual patients were referred to the programs for a duration of 12 weeks and were followed up by HF nurses, and physical and occupational therapists. At 1-month follow-up, all participants completed the same questionnaire as at baseline. Due to the scope of the study, the primary researcher collected all data, as blinding was not possible. All study procedures were approved by the relevant hospital and university human research ethics committees. The intervention was also made available to the control group after completion of the research.

### Intervention

The pedagogical principals underpinning the Web-based app were based on two key elements of self-efficacy theory [15,16]: role modelling and mastery experience. Helping adults to improve their self-efficacy is a powerful and central factor in increasing chronic disease self-care [5,17]. Patients with greater self-efficacy have been found to be more willing to learn and commit to achieving goals, whereas low self-efficacy has been associated with task avoidance [18]. We anticipated that the use of informational sources such as role modelling and mastery experience would help patients to increase their self-efficacy. Role modelling can take the form of observing the actions of others [18]. As such, we created female and male avatars to resemble role models and to help users engage with educational materials on the website. We encouraged mastery experience by asking participants to monitor and record their signs and symptoms related to HF fluid overload. The daily weight measurements and HF symptoms were able to be recorded to show trends over time. We used a further source of self-efficacy, verbal persuasion, through weekly emails to each participant.

We developed the Web-based app based on feedback from three groups: HF experts, an information technology team, and HF patients. The role of the HF expert panel was to ensure integrity of the content according to evidence-based guidelines, applicability of study instruments, appropriate language and images, and appropriate user interfaces. HF patients provided their feedback and recommendations on the content, ease of navigation, and user friendliness. We developed the Web-based educational materials based on the National Heart Foundation of Australia and the Cardiac Society of Australia and New Zealand chronic HF guidelines [3]. The website was password protected, and both HF patients and their health care professionals had access to content including interactive HF teaching tools, self-care tools, a chart for recording daily measures, and self-care questionnaires.

#### Teaching Tools

This section consisted of educational topics including an explanantion of HF (with animations), HF signs and symptoms, daily weighings (related to fat and fluid differentiation), suggestions for healthy eating and being active, methods to avoid salty foods and tips for reading food lables, and taking appropriate action when symptoms were exacerbated.

#### Self-Care Tools

To reinforce self-monitoring, patients could enter daily clinical data including daily weight and any signs of fluid retention such as tight shoes or socks.

#### My Chart

This tool allowed patients to visually monitor their daily weight and severity of HF symptoms through simple graphs. In addition, health care professionals were able to monitor their patients’ self-reported data by accessing their charts.

#### Self-Care Questionnaires

The final section was designed to help patients assess their knowledge and self-care skills over time. It included questionnaires such as the Self-Care of Heart Failure Index (SCHFI) and the Dutch Heart Failure Knowledge Scale (DHFK).

### Outcome Measures

In addition to baseline demographics, questionnaires assessed the primary outcomes including HF knowledge, self-care, and self-efficacy. The DHFK [19] consists of 15 multiple-choice items concerning HF in general (4 items), HF treatment (6 items on diet, fluid restriction, and activity), and HF symptoms and symptom recognition (5 items). The scale has a minimum score of 0 (no knowledge) and a maximum score of 15 points (optimal knowledge). It is a frequently used measure of HF knowledge and has been validated in previous research evaluating educational intervention [1].

We measured self-care skills using the SCHFI version 6.2 [20,21]. This tool has 21 items, which are scored as three subscales: maintenance (symptom monitoring and adherence behaviors performed to prevent HF exacerbation), management (patients’ abilities to recognize symptoms when they occur, treatment implementation in response to symptoms, and treatment evaluation), and confidence (measures task-specific self-efficacy behaviors to manage the process of self-care) [5]. The management subscale was answered only if the patient reported having trouble breathing or ankle swelling in the past 4 weeks. Each standardized subscale score ranged from 0–100, with a score of ≥70 or more indicating adequate self-care [21].

We also used the 6-item Self-Efficacy for Managing Chronic Disease Scale (SEMCD) [22] as a general measure of chronic disease self-efficacy. It covers several domains relevant to chronic disease self-care, including symptom control, role-function, emotional functioning, and communicating with physicians. Each item is rated on a 10-point scale ranging from “not at all confident” (1) to “totally confident” (10). A mean score is calculated, with higher scores indicating higher perceived self-efficacy.

### Data Analysis

We analyzed data using IBM SPSS Statistics version 21 (IBM). We compared baseline characteristics using Fisher exact and *t* tests. Descriptive statistics were examined for all variables, and pre-post change scores were calculated for outcome measures. Given the small sample size and nonnormal distribution of outcome variables and to analyze between-group differences over time, we compared change scores using Mann-Whitney *U* tests. We considered *P*<.05 to be significant and report effect sizes where possible.

## Results

### Study Recruitment and Baseline Characteristics

**Figure 1:**
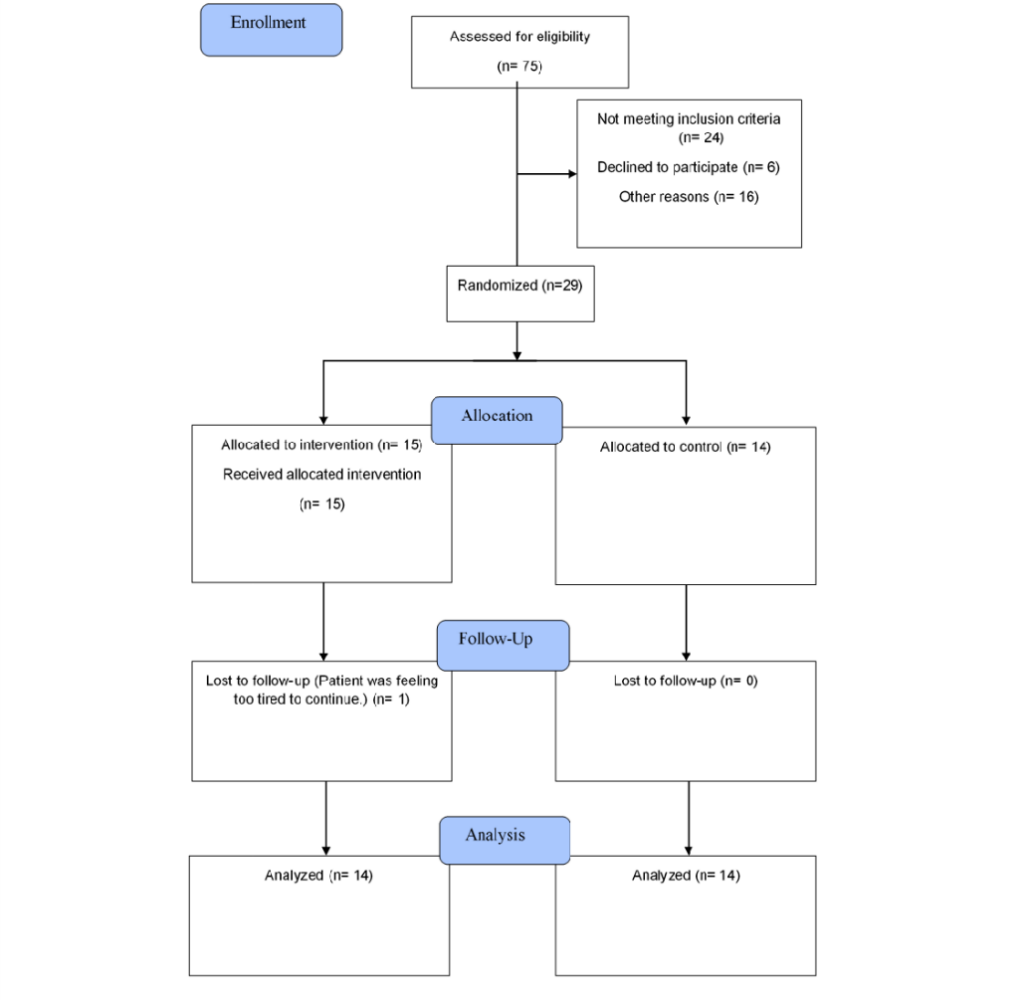
Consort study flow chart.

Of 75 HF patients who were assessed for eligibility, 29 agreed to participate and were enrolled into the study (see Figure 1). Table 1 shows the baseline demographic and clinical characteristics of the 28 patients who participated in the pilot study. One participant dropped out. Of the total sample, the majority were men (n=22, 79%) and the mean age was 60.8 (SD 11.9) years. The mean self-reported disease duration was 4.0 (SD 6.9) years. Most participants were asymptomatic (class I, n=14, 50%) or had mild HF (class II, n=12, 43%) according to the NYHA functional classification. Comparisons showed no significant differences between the intervention and control groups at baseline.

**Table 1:** Baseline characteristics of participants in a pilot study of a Web-based self-management intervention for chronic heart failure.

Characteristic		Control (n=14)	Intervention (n=14)
Age in years, mean (SD)		60.0 (14.0)	61.7 (9.9)
**Sex, n (%)**			
	Male	11 (79)	11 (79)
	Female	3 (21)	3 (21)
**Marital status, n (%)**			
	Married	8 (57)	9 (64)
	Widowed, divorced, or never married	6 (43)	5 (36)
**Living arrangement, n (%)**			
	Live alone	6 (46)	5 (36)
	Live with others	7 (54)	9 (64)
**Level of education, n (%)**			
	<12 years or high school diploma	7 (50)	8 (57)
	Some college/associate degree, bachelor’s degree, or postgraduate degree	7 (50)	6 (43)
**Total household income, n (%)**			
	≤A$40,000/year	4 (29)	9 (64)
	≥A$70,000/year, or do not know, or refused	10 (71)	5 (36)
**Overall perceived health, n (%)**			
	Excellent, very good, or good	7 (50)	7 (50)
	Fair or poor	7 (50)	7 (50)
**Medication treatment**			
	Angiotensin-converting enzyme inhibitor	10 (71)	10 (71)
	Angiotensin II receptor blocker	4 (29)	2 (14)
	Beta-blocker	14 (100)	11 (79)
**New York Heart Association classification**			
	Class I	8 (57)	6 (43)
	Class II	6 (43)	6 (43)
	Class III	0 (0)	2 (14)
Ejection fraction, mean (SD)		33.7 (8.9)	33.4 (8.51)
Duration of heart failure in years, mean (SD)		3.0 (4.8)	4.9 (8.5)

### Heart Failure Knowledge

Table 2 compares the change in knowledge scores for the intervention and control groups. As the maximum possible knowledge score is 15, the baseline mean scores of 12.5 (SD 1.1) for the intervention group and 12.2 (SD 2.4) for the control group indicated that participants had relatively high levels of HF knowledge at the outset. While change scores at 1 month showed slight improvement in HF knowledge, there was no significant difference between the intervention and control groups (*U*=91.5, *P*=.75), with a negligible effect size (*d*=0.06).

**Table 2:** Change score analysis: change in scores from baseline preintervention (pre) to post intervention (post).

Scale	Intervention group (n=14)		Pre-post difference, mean (95% CI)	Control group (n=14)		Pre-post difference, mean (95% CI)	Mann-Whitney *U* test	*P* value	Effect size (*d*)
	Pre, mean (SD)	Post, mean (SD)		Pre, mean (SD)	Post, mean (SD)				
DHFK^a^	12.5 (1.1)	12.9 (1.3)	0.35 (–0.67 to 1.3)	12.2 (2.4)	12.8 (1.7)	0.57 (–0.05 to 1.2)	91.5	.75	0.06
SCHFI^b^ (maintenance)	61.9 (20.9)	71.9 (15.1)	9.9 (–3.6 to –23.6)	70.2 (17.4)	66.6 (17.7)	–3.5 (–10.3 to 1.3)	59.5	.07	0.32
SCHFI (confidence)	67.1 (20.3)	73.0 (18.0)	3.8 (–4.3 to 12.0)	65.5 (21.4)	69.9 (19.2)	3.8 (–4.3 to 12.0)	89.5	.94	0.16
SEMCD^c^	7.3 (1.6)	8.0 (1.7)	0.70 (–0.54 to 1.9)	7.1 (1.4)	7.4 (1.5)	0.35 (–0.21 to 0.92)	87.0	.61	0.37

^a^DHFK: Dutch Heart Failure Knowledge Scale.

^b^SCHFI: Self-Care of Heart Failure Index.

^c^SEMCD: Self-Efficacy for Managing Chronic Disease Scale.

### Self-Care

As presented in Table 2, change scores showed an improvement in the SCHFI maintenance subscale in the intervention group to above the minimum level of self-care adequacy (mean difference 9.9, 95% CI –3.6 to –23.6), whereas the control group scores decreased over time (mean difference –3.5, 95% CI –10.3 to 1.3). While the effect size suggests an important trend (*d*=0.32), given the small sample size it did not reach statistical significance (*U*=59.9, *P*=.07). In addition, while we observed small improvements in the SCHFI confidence subscale, we found no significant between-group differences (*U*=89.5, *P*=.94). As answers to the management subscale were indicated by only 5 patients, we did not compare this subscale.

### Self-Efficacy

Table 2 shows that both groups also reported high levels of self-efficacy at baseline. Change scores indicated an improvement among the intervention group (mean difference 0.70, 95% CI –0.54 to 1.9, *d*=0.37), although this was not statistically significant (*U*=87.0, *P*=.61).

### Use of the Intervention

The intervention group accessed the website on average 16.8 times during the 1-month intervention period. Of the 14 participants, 7 (50%) accessed the intervention site every day, 4 (28%) had no record of access, 11 (78%) accessed the website from home and 3 (22%) through a mobile phone. The most reviewed section of the website was “How do I learn to read food labels?” followed by “How do I reduce salt in my daily meal?,” “What is the difference between fat and fluid weight gain?,” and “How do I become more physically active?” Based on personal data recorded, 7 participants entered their data every day, 1 participant on 25 days, 1 on 12 days, and 1 on 4 days; 4 participants did not enter any data.

To explore a possible dose-response relationship, we ran correlations between participants’ change scores and frequency of usage of the website. Within the intervention group, Spearman correlations between frequency of usage of the intervention and outcome change scores suggested greater use was associated with higher knowledge (ρ=.34), SCHFI subscales (ρ range .40–.43), and self-efficacy scores (ρ=.45).

## Discussion

### Principal Findings

In this pilot study, we developed and investigated the feasibility of a Web-based self-care intervention for HF outpatients. Overall, results showed that the intervention did not improve patients’ outcomes at 4 weeks’ follow-up. While there was a trend toward improved self-care and self-efficacy in the intervention group for behaviors such as symptom monitoring and strategies to prevent HF exacerbation, given the small sample size, the results were not statistically significant. In contrast to previous studies [10,11] we also found that a Web-based educational intervention was challenging for older HF patients, and usage was low. Despite face-to-face training and email reminders, only 50% of the sample accessed the site daily and 28% had no record of access.

### Comparison With Prior Work

This study differed in important ways from previous intervention studies of Web-based HF self-care [11,12], which may account for the negative findings. Our cohort had shorter disease duration and less severe HF according to the NYHA classification than in previous studies [11,12]. The follow-up period was relatively short to examine changes in patient outcomes, and we acknowledge that a minimum of 6 months’ follow-up is recommended in the literature [12,23]. There were also differences in the scope of the intervention. The educational content of the website was at a basic level and focused on self-care skills. The standard face-to-face patient education received by both groups in the HF clinic, therefore, may not have been comprehensive enough to diminish the effect of the Web-based intervention. Our primary outcome measures of self-care skills and self-efficacy differed from previous studies, which focused on HF knowledge, symptoms, and quality of life [11,12]. Nonetheless, using validated measures was a strength of this study. Yet, because most of the participants in the sample were asymptomatic, we did not compare the management subscale of the SCHFI. In addition, as the maximum score for the DHFK questionnaire was 15 and a score >10 indicated adequate knowledge [19], the high knowledge scores of participants at baseline (mean 12.5, SD 1.1) likely created a ceiling effect.

Notwithstanding the above limitations, our study findings are consistent with a body of literature that points to mixed evidence of the effectiveness of Web-based self-care interventions for chronic disease [13,24]. Despite the development of a plethora of Internet-based interventions over recent decades, systematic reviews have not shown any benefit of Web-based self-care interventions for chronic disease [25,26]. Moreover, meta-analyses have not demonstrated a significant benefit in the use of Web-based interventions when combining samples [27,28]. This is attributed, in the first instance, to issues of methodology and variability in research design [13,29-31]. There are significant differences in configuration (eg, educational materials, asynchronous discussion, live conferencing), instructional methods (eg, practice exercise, cognitive interactivity), and presentation [13]. These also extend to types of assessments, study populations, the etiology of symptoms, and times of intervention [27,32]. A further and related issue is a lack of appropriate theoretical frameworks [33,34]. Many studies are missing the theoretical rationale for multiple assessments and how these informed the development of the Web-based interventions. The authors of a previous study [33] reported that a limited number of interventions applied evidence-based theory and, more recently, a meta-analysis of research on reducing blood pressure with Internet-based interventions [34] emphasized that a priority for future research is to design and evaluate interventions according to theoretically grounded hypotheses.

In contrast to other kinds of educational intervention studies in HF patients [1], the role of theory in developing Internet-based interventions has been largely disregarded. While precise measures of key constructs and outcomes and well-defined associations improve research precision, theory is needed to inform the choice of study design and to enhance an understanding of causal relationships [35]. Robust theory is also critical in identifying the effectiveness of the specific components of the interventions and optimizing their intensity [35-37]. Key theoretical constructs and associations should therefore be applied in efficacy trials, tests of effectiveness, and adoption and sustainability studies. There is some evidence for the use of Bandura’s self-efficacy theory as the most important element in developing self-care interventions [7].

### Implications for Future Research

This pilot study presented a fundamental phase of the development of a Web-based self-care intervention for patients with HF that can be used to inform future research. To examine the effectiveness of the proposed Web-based intervention, a larger, adequately powered randomized controlled trial is needed. Longer follow-up periods at 3, 6, and 12 months are required to examine clinically meaningful change over time. In addition, we recommend that researchers actively support older HF patients to engage with Web-based interventions through face-to-face education sessions for basic computer and Internet skills. Greater resources may improve adherence and resolve possible barriers or difficulties in using Web-based self-care tools.

Well-designed educational strategies grounded in theory and contemporary evidence are crucial in improving HF self-care and patient outcomes [38]. More comprehensive educational content is needed, including nutrition and diet, physical activity, effects of alcohol and smoking, medications and their side effects [5], as well as psychosocial and emotional issues [1,39]. Moreover, as Tomita et al [12] argued, interventions should target improved physical activity and provide practical advice about appropriate types and levels of exercise. This would include walking, breathing, stretching, range of motion, and upper and lower extremity strength training. For better outcomes and in order to encourage HF patients to maintain healthy lifestyles, interventions would also incorporate more information on recommended diet and nutrition [12].

To strengthen the efficacy of the Web-based intervention, we recommend drawing on the sources and mediators of self-efficacy theory. Feedback, goal setting, videos of peer storytelling, monitoring tools such as for blood pressure, daily weight, and physical activities, and diaries are some examples of self-efficacy information sources [40]. We strongly recommend using a combination of the four primary information sources in Bandura’s work to promote a stronger sense of self-efficacy and a greater willingness to undergo behavioral change and thus produce optimal results [41]. There are examples [6,7] of previous studies that have used the four primary dimensions of self-efficacy to enhance self-care in adults with chronic diseases. The primary focus of these interventions was the provision of efficacy-based information sources, including the mastery of performance accomplishments, role modelling (vicarious learning), social persuasion (verbal encouragement), and the interpretation of physiological and emotional responses [7].

### Limitations

Recruitment was slow in part due to HF patients’ lack of interest in the intervention, Internet access, or computer skills. Among the HF patients who were invited to participate in the study, 26 were not eligible based on the study inclusion and exclusion criteria. This exploratory pilot study was confined to an HF management program provided at a single-hospital HF service and university health clinic. To participate, it was essential that HF patients had access to the Internet at home or workplace or via a mobile phone. Although a large proportion of the HF population did have access to the Internet, a significant minority (22 participants) did not. As a result, our study was based on a small sample size, which limited statistical power. Finally, the study follow-up was relatively short for the examination of the Web-based intervention on patient knowledge, self-care, and self-efficacy.

### Conclusion

This study examined the feasibility of a Web-based self-care intervention in improving HF knowledge, self-care, and self-efficacy. Although preliminary, our findings are consistent with current literature that demonstrates negative to small effects of Web-based self-care interventions in chronic disease. Larger, theoretically informed, and more comprehensive studies are needed to support or refute this proposition. Nonetheless, the challenges of this study and lessons learned give some support to the argument that Web-based programs, as discrete interventions, will not be the remedy for the cost and social burden of chronic illness.

## Abbreviations

DHFK: Dutch Heart Failure Knowledge Scale
HF: heart failure
NYHA: New York Heart Association
SCHFI: Self-Care of Heart Failure Index
SEMCD: Self-Efficacy for Managing Chronic Disease Scale
